# Risk spillover networks in financial system based on information theory

**DOI:** 10.1371/journal.pone.0252601

**Published:** 2021-06-18

**Authors:** Weibo Li, Wei Liu, Lei Wu, Xue Guo

**Affiliations:** 1 School of Economics, Wuhan Textile University, Wuhan, Hubei, China; 2 School of Mathematics and Compute Science, Wuhan Textile University, Wuhan, Hubei, China; Aristotle University of Thessaloniki, GREECE

## Abstract

Since the financial system has illustrated an increasingly prominent characteristic of inextricable connections, information theory is gradually utilized to study the financial system. By collecting the daily data of industry index (2005-2020) and region index (2012-2020) listed in China as samples, this paper applies an innovative measure named partial mutual information on mixed embedding to generate directed networks. Based on the analysis of nonlinear relationships among sectors, this paper realizes the accurate construction of “time-varying” financial network from the perspective of risk spillover. The results are presented as follow: (1) interactions can be better understood through the nonlinear networks among distinct sectors, and sectors in the networks could be classified into different types according to their topological properties connected to risk spillover; (2) in the rising stage, information is transmitted rapidly in the network, so the risk is fast diffused and absorbed; (3) in the declining stage, the network topology is more complex and panic sentiments have long term impact leading to more connections; (4) The US market, Japan market and Hongkong market have significant affect on China’s market. The results suggest that this nonlinear measure is an effective approach to develop financial networks and explore the mechanism of risk spillover.

## Introduction

Over the past decades, the network theory has been applied to understand and explain some real-world systems, consisting of a large number of components that interact with each other [1–4]. The network, based on the connectivity, is constructed by linking any two nodes when exploiting significant information between them and the topological properties in real-world social networks differ from the random graph or the regular graph [1, 2]. When it comes to the study of financial networks, both theoretically and empirically, it contributes to the research of the financial risk since the financial system has illustrated an increasingly prominent characteristic of inextricable connections [2, 5]. The reason for this is that with description of fluctuation interdependence of the asset prices, the impact of individual characteristics in the network, at the micro level, is the root of the system fluctuation and becomes system risk through cumulative fluctuations [5–7]. Risk spillovers, corresponding to crisis events, usually demonstrate clear burst associated volatility dependence.

The literature of this study has covered the financial networks in the description of financial risk spillover. The network structure can magnify system risk [8–10]. According to Curme et al. (2015), the stock network has different topological properties in different periods, the efficiency and instability have been growing in the stock market [11]. As it is studied by Han et al. (2017), the network has different structures around the financial crisis and may be fragile to targeted attacks [12]. From the contagion pattern, the risk center of system could be discovered [13]. Based on the studies above, we can find that these networks, correlation based, are developed by filtering the complexity of financial dependencies [5, 14–16].

Two types of networks are mainly constructed based on the sort of connections, in the form of undirected and directed. As for undirected networks are developed by symmetric correlations with extraction of essential information from the associated networks. There are three major methods to get the undirected graph by filtering the crucial information from a complete graph, namely the minimum spanning tree(MST) [15, 17], planar maximally filtered graph(PMFG) [2] and threshold graph [15, 18]. To be more specific, MST extracts a general hierarchical structure [19] and PMFG favors connections formed by cliques [20] while threshold graph is determined by a threshold value [15]. According to Onnela et al. (2004), he constructed a stock network using the stock trading price data of the New York Stock Exchange and found that the stock network showed more obvious cluster characteristics where the important nodes contained most of the effective information [14]. Tumminello et al. (2005) analyzed the formed clusters based on a filtered network, which keeps the same hierarchical tree to the corresponding MST [20]. Birth et al. (2015) discovered that less unconnected links were found in the period of recovery than the period of crisis [18]. In light of the findings above, this kind of networks do not contain the direction information of links, but the direction of information spillover in stock market is very important. We can find that undirected network lacks clear economic meaning.

Compared with undirected graph, directed networks have clearer economic significance. The Granger causality test is utilized to build directed links through the causal property [21]. Yao et al. (2016) confirmed that the causal property can explain the routes of risk transmitting between banks, securities, hedge funds and some other financial agencies [22], and the Granger causality test considers the pairwise correlation between variables. Diebold and Yilmaz(2014) analyzed connectedness of major US financial institutions’ stock return volatility on the basis of variance decompositions [23]. The measure of impulse response is also applied. According to Alter and Beyer (2014), they captured changes of interdependence among sovereigns and banks over time and potential systemic risk increased with a clear upward trend of growing interdependence between banks and sovereighs [24]. These networks are constructed through vector autoregressive model, the limitation of which in this study is that it can only suit the low dimensional model. So Demirer et al. (2018) proposed to use Least Absolute Shrinkage and Selection Operator (LASSO) to reduce the dimension when constructing high dimensional network [25], though LASSO performs poorly when the variables are highly correlated.

With the capital and business exchanges reaching a certain density and depth, the financial system has showed more complicated associations [26]. Considering the limitation of vector autoregressive model, recently, this study makes several attempts to extend the linear to nonlinear and extend the low dimension to high dimension have been proposed. Hence, information-theoretic approaches are increasingly applied for network construction since they can measure nonlinear interrelationships properly [27–31]. The mutual information has been used measure the degree of interaction between each variable and its parent variables [27]. In financial system, the mutual information has also been used to develop stock network to detect the violent fluctuation of stock prices [32], which also suits analysis of high-frequency data [33]. Based on Sandoval(2014), the study by using transfer entropy(TE), there are causal relationships between stocks to assess influences within financial sectors and the network is very different from correlation-based network [26, 34]. but the researches on indirect coupling are few when the interdependence between two variables under the influences of other variables, namely confounding variables. Considering the influences of confounding variables, partial transfer entropy(PTE) is multivariate extension of TE [35, 36]. Also partial symbolic transfer entropy(PSTE) performs well with the non-stationarity time series but not effective with only linear couplings [37]. And conditional mutual information(CMI) [38, 39] are developed to discover nonlinear correlations with complex underlying properties. It is worthy mentioning that these measures are effective on low-dimensional systems but less sensitive to high-dimensional systems [40].

In order to overcome the limitations of bivariate causality and problematic for high dimensional data sets, the measure of partial mutual information on mixed embedding(PMIME) can be used to analyze couplings among multi-dimensional time series [41, 42]. PMIME is a sort of information theoretic measure based on transfer entropy, frequently used to process a large number of observed variables as well as high dimensional variables but fewer spurious causal effects, which outperforms standard linear conditonal Granger causality index and PTE [41, 43]. And PMIME is not rely on significance test [43, 44].

In this perspective, our object is to specify the nonlinear and multivariate coupling among different sectors in China’s security market and describe the risk contagion in the resulting networks by using PMIME. The following part of this paper is to be presented as follows: Section 2 briefly presents the causality measure of PMIME and develops the stock networks based on the measure. Section 3 introduces the financial data an related procession. Section 4 reports the results and related discussion, and conclusion is presented in section 5 afterwards.

## Methodology

### Approach of PMIME

PMIME is an information-based measure, which can effectively discover the connectivity patterns of multivariate systems. Suppose that a dynamical system could be reconstructed by an univariate or multivariate time series, to form a state space in a way, more information about the original dynamics can be represented. In a system represented as {*X*_1_, *X*_2_, …*X*_*m*_}, a subsystem observed through *X*_1_ drives a direct effect on response subsystem *X*_2_ under the influence of other subsystems *Z* = {*X*_3_, …*X*_*m*_}, where the observed variables in *Z* are referred to as confounding variables. The main idea of this measure is to reconstruct a joint state space through embedding lagged variables of *X*_1_, *X*_2_, *Z* to explain the evolution of *X*_2_.

The key factor of this measure is identification of the embedding dimension of variables and their lags. Derived from Takens’ embedding theorem [45], the uniform embedding scheme for a given time series ${\left\{x_{t}\right\}}_{t=1}^{N}$ is described as *X* = (*x*_*t*_, *x*_*t*−*τ*_, …, *x*_*t*−(*p*−1)*τ*_), where *p* is the number of delayed components in {*x*_*t*_} and *τ* is the delayed time. Similarly, for multivariate time series ${\left\{x_{i,t}\right\}}_{t=1}^{n}$, *i* = 1, …, *m*, the uniform reconstructed state space vector is extended as the form

$$\begin{array}{c} x_{t}=\left(x_{1,t},\ldots ,x_{1,t-\left(p_{1}-1\right)\tau_{1}},x_{2,t},\ldots x_{m,t-\left(p_{m}-1\right)\tau_{m}}\right) \end{array}$$ (1)

with the embedding dimension vector ***p*** = (*p*_1_, …, *p*_*m*_) which is the indication of components from each time series, and $\sum_{i=1}^{m}p_{i}$ is the dimension of system. In addition, a time delay vector ***τ*** = (*τ*_1_, …, *τ*_*m*_), varied *τ*_*i*_ for each time series, depends on different cases. The future of *x*_*i*,*t*_ is generally defined as $x_{i,t}^{T}=\left(x_{i,t+1},\ldots ,x_{i,t+T}\right)$. A major issue in the multi-variate state space is identification. For the univariate time series, the embedding vectors are uniquely defined by a given value *τ*. But multivariate data would have different levels of *τ*, and all possible combinations of components for the determination of the optimum embedding would be computationally difficult when the embedding dimension and the number of time series get large, although non-uniform scheme for multivariate embedding may filter the redundant information than the embedding with fixed lags. For the empirical analysis in this study, we set *τ*_*i*_ = 1, *i* = 1…*m*. And Vlachos and Kugiumtzis(2010) proposed a measure to confirm the minimum adequate dimension to sufficiently detect interdependence [46].

The embedding scheme is described as follow: Let ***D*** is a candidate set with the maximum lags *L*_*i*_ for each time series, *i* = 1, …, *m*,

$$\begin{array}{c} D=(x_{1,t},x_{1,t-1},\ldots ,x_{1,t-L_{1}},x_{2,t},\ldots x_{m,t-L_{m}}) \end{array}$$ (2)

and the embedding vector would be selected from set ***D***. With ideas developed for the state space reconstruction from multi-variate time series, the reconstructed vector would satisfy two properties that its components should be least dependent to each other and it will explain the dynamics of the system best. The proposed scheme starts from an empty vector ***d***_**0**_ = ∅. For $x_{1,t}^{T}$, We obtain the component in ***D*** being most correlated to it by estimation of mutual information, $d_{t}^{1}={\text{argmax}}_{d\subset D}I\left(x_{1,t}^{T};d_{t}^{1}\right)$, then we confirm $d_{t}^{1}=\left[d_{t}^{1}\right]$. The iterative process would be repeated, a new component would be added to the existing vector at each step. Suppose we have selected *j* − 1 components $d_{t}^{j-1}$, $d_{t}^{j}$ would be accepted significantly and proceeded to the next embedding cycle if

$$\begin{array}{c} I\left(x_{1,t}^{T};d_{t}^{j}|d_{t}^{j-1}\right)/I\left(x_{1,t}^{T};d_{t}^{j},d_{t}^{j-1}\right)\geq b \end{array}$$ (3)

*b* = 1 − *α*, otherwise $d_{t}^{j}=d_{t}^{j-1}$.

Depending on the form of ***D*** and $x_{1,t}^{T}$, this measure can be used for reconstruction of multi-variate state space. However, it is difficult to estimate the mutual information of driving vectors and response vector because of multi-dimension, the nearest neighbors method [47] is applied, instead of the binning estimation [32]. Given the obtained set ***D***, PMIME has been developed as the mixed embedding vector which can best describe the future of *X*_1_, defined as follow:

$$\begin{array}{c} P_{X_{2}\to X_{1}|X_{3},\ldots ,X_{m}}=\frac{I(x_{1,t}^{T};d_{t}^{x_{2}}|d_{t}^{x_{1}},d_{t}^{x_{3}},\ldots d_{t}^{x_{m}})}{I(x_{1,t}^{T};d_{t})} \end{array}$$ (4)

The numerator of Eq (4) is the conditional mutual information of the future response $x_{1,t}^{T}$ and the part of embedding vector developed by lags of the driving variable $d_{t}^{x_{2}}$ based on the rest part of embedding vector. Then it is normalized by the mutual information of the future response $x_{1,t}^{T}$ and the whole embedding vector. So *P* takes value in [0, 1], where there exists nonlinear relationship with positive value. The Eq (4) describes a subsystem that driving vector *X*_2_ has a certain effect to the response vector *X*_1_ based on the embedding vector.

Test for nonlinear relationship using TE and PTE have been also suggested in terms of entropies. The TE quantifies the amount of information explained in *X*_1_ at *T* steps ahead from *X*_2_ based on the concurrent state of *X*_1_, expressed as

$$\begin{array}{c} TE_{X_{2}\to X_{1}}=I\left(x_{1,t+T};x_{2,t}|x_{1,t}\right)=H\left(x_{1,t+T}|x_{1,t}\right)-H\left(x_{1,t+T}|x_{2,t},x_{1,t}\right) \end{array}$$ (5)

In the Eq (5), *I*(*x*_1,*t*+*T*_;***x***_**2**,***t***_ **|** ***x***_**1**,***t***_) is the conditional mutual information of *x*_1,*t*+*T*_ and ***x***_**2**,***t***_ accounting for *x*_1,*t*_, and *H*(*x*_1,*t*+*T*_|***x***_**1**,***t***_) is the conditional entropy of *x*_1,*t*+*T*_ accounting for ***x***_**1**,***t***_. The PTE is the extension of the TE by estimating the nonlinear relationship of *X*_2_ to *X*_1_ based on the rest variables in the system, namely *Z*

$$\begin{array}{c} PTE_{X_{2}\to X_{1}|Z}=H\left(x_{1,t+T}|x_{1,t},z_{t}\right)-H\left(x_{1,t+T}|x_{2,t},x_{1,t},z_{t}\right) \end{array}$$ (6)

The comparative analysis between PMIME and PTE will be done in the later section.

### Measures of network topology

Based on the estimated analysis of PMIME, we can get a filtered adjacency matrix represented as *A*_*m*×*m*_ = (*a*_*i*,*j*_) with *i*, *j* = 1, …*m*, where the element *a*_*i*,*j*_ indicates causality from node *i* to *j* and *m* stand for the number of nodes in the network. In this study, all results are extracted from binary directed networks, where there is a connection from *i* to *j* if *a*_*i*,*j*_ ≠ 0. A variety of statistics have been applied to analyze the network topology. Considering the mechanism of market risk spillover, we will introduce several measures to study the network topology.

#### Node degree

Node degree, the number of corresponding connections associated with a node, is a fundamental topology property of network. In the directed network, the degree can be classified into outdegree and indegree according to the direction of associations, and they are not symmetric. Outdegree of a certain node is defined as connections from it to other nodes while indegree is vice versa, respectively characterizing the influential and influenced effect. In this study, outdegree of the node can explain the extent of risk diffusion, correspondingly, indegree can describe the risk absorption.

In order to determine the importance and property of a node, some measures are proposed. For example, degree ratio has been proposed to rank the nodes, and discriminate influential nodes and influenced nodes [48]. From the perspective of risk propagation, we propose degree centrality to discriminate different properties of nodes, namely the outdegree centrality index and indegree centrality index. The outdegree centrality index of node *i* is calculated as the sum of PMIME value from it to other nodes and normalized.

$$\begin{array}{c} \begin{array}{cc} & OC_{i}=\sum_{j=1}^{m}a_{ij},\\ & OCI_{i}=\frac{OC_{i}-Min\left\{OC_{i}\right\}}{Max\left\{OC_{i}\right\}-Min\left\{OC_{i}\right\}}, \end{array} \end{array}$$ (7)

where *OC*_*i*_ is outdegree centrality of node *i* and *OCI*_*i*_ is its normalized value. Similarly, the indegree centrality index of node *i* is calculated as the sum of PMIME value from other nodes to it and normalized.

$$\begin{array}{c} \begin{array}{cc} & IC_{i}=\sum_{j=1}^{m}a_{ji},\\ & ICI_{i}=\frac{IC_{i}-Min\left\{IC_{i}\right\}}{Max\left\{IC_{i}\right\}-Min\left\{IC_{i}\right\}}. \end{array} \end{array}$$ (8)

Here, *IC*_*i*_ is indegree centrality of node *i* and *ICI*_*i*_ is its normalized value.

#### Distance

In order to investigate the information transition within the network, the distance *d*_*i*,*j*_ is defined as the length of the shortest path from node *i* to *j*, which is calculated as the minimum connections to reach between two nodes. The mean distance of the whole network is $\sum_{i\neq j}\frac{d_{i,j}}{N(N-1)}$ (exclusion of unreachable node pairs). Usually, the average distance can describe the efficiency of information diffusion within the network.

#### Betweenness

Betweenness is an important global feature quantity in the network. If node *i* is passed by many other shortest paths, it means that the node is very important in the network. Its importance or influence can be expressed by $B_{i}=\sum_{j\neq i\neq l}\frac{n_{jl}\left(i\right)}{n_{jl}}$, where *n*_*jl*_ is the number of shortest paths between nodes *j* and *l* and *n*_*jl*_(*i*) is the number of the shortest path between nodes *j* and *l* through node *i*. It can be seen that the betweenness of *i* is the ratio of the number of nodes passing through the shortest paths in the network. Although the degree of some nodes is very small, it may be an intermediary between two communities. If this node is removed, the connection between the two communities will be interrupted.

#### Clustering coefficient

The clustering coefficient of the graph reflects the characteristics of the small world. For a node *i*, its degree is *k*_*i*_, which means *k*_*i*_ nodes connect to it. Then the amount of possible connections of this node is *k*_*i*_(*k*_*i*_ − 1)/2, while the actual number of connections is *E*_*i*_. The clustering coefficient of node *i* is the ratio of actual number of connections and possible number of connections, called *CC*_*i*_. The formula is represented as $CC_{i}=\frac{2E_{i}}{k_{i}\left(k_{i}-1\right)}$. Then the average clustering coefficients of all nodes in the network is called the average clustering coefficient of the network, recorded as *CC*, and 0 ≤ *CC* ≤ 1. When *CC* = 1, any two nodes in the complex network are connected and become a complete network. When *CC* = 0, all the nodes in the network are isolated.

#### Clique formation

Based on the clustering coefficient, the clustering structure in network is that nodes in the network can be divided into several groups with dense intra-group and sparse inter-group connections. Clustering is an essential symbol in financial system, which is investigated in terms of the clique. A clique, represented as *K*_*q*_ (*q* ≥ 3), is generally fully connected subgraph composed of three or more nodes [49]. For example, *K*_3_ contains three nodes, any two of which are connected to each other. Nodes in the same clique would have stronger mutual influences than the nodes outside of this clique.

## Data and variables

### Data sources

This study makes empirical analysis of industry index and region index in the stock market. A sample for empirical analysis needs to be chosen properly as all indexes have the same consecutive trading period. Therefore, we construct networks with two data sets, which consist of daily prices of industry indexes and region indexes in China Stock Exchange. According to ShenWan Industry classification standard, there are 28 sectors including Shery(SY), Mining(MI), Chemicals(CHE), Steel(STE), Non-ferrous Metals(NFM), Electronics(ELE), Household Appliances(HA), Food & Beverage(FB), Textile and Garment(TG), Light Industry Manufacturing(LIM), Pharmaceutical Biology(PB), Utilities(UT), Transportation(TS), Real Estate(RE), Commercial Trade(CT), Leisure Services(LS), Composite(CP), Construction Materials(CM), Construction Decoration Materials(CDM), Electrical Equipment(EE), National Defense & Military(NDM), Computer(CPT), Media(MED), Communications(CMM), Banks(BA), Non-Bank Financial(NBF), Auto(AU) and Machinery & Equipment(ME). The period of sample spans from June 01, 2005 to May 29, 2020. When it comes to region indexes, they include 36 regions composed by CNI Shenzhen Innovation DEMO(SZI), Shenzhen Enterprises Composite(SZEC), Yangtze, Zhujiang, Bohai, Anhui A Share, Beijing A Share, Fujian A Share, Gansu A Share, Guangdong A Share, Guangxi A Share, Guizhou A Share, Hainan A Share, Hebei A Share, Henan A Share, Heilongjiang A Share, Hubei A Share, Hunan A Share, Jilin A Share, Jiangsu A Share, Jiangxi A Share, Liaoning A Share, Inner Mongolia A Share(Inner Mongo), Ningxia A Share, Qinghai A Share, Shandong A Share, Shanxi A Share, Shaanxi A Share, Shanghai A Share, Sichuan A Share, Tianjin A Share, Tibet A Share, Xinjiang A Share, Yunnan A Share, Zhejiang A Share and Chongqing A Share. This process of data collecting started from December 29, 2012 and ended on May 29, 2020 because most region indexes were disclosed since then. It is noted that during the sample period, China security market experienced several notable ups and downs, so we divide some subsamples in consideration of the rise and fall of market index in order to compare the diverse topologies of stock networks at different stages. The data have been collected from wind database.

Since we attempt to figure out the relationship between stock price fluctuation and market risk, the daily return volatility should be applied to replace closing price. In order to contain more information, we usually refer to formula proposed by Garman and Klass to obtain the daily return volatility [50],

$$\begin{array}{cc} \sigma_{it}^{2}= & 0.511{\left(h_{it}-l_{it}\right)}^{2}-0.019[\left(c_{it}-o_{it}\right)\left(h_{it}+l_{it}-2o_{it}\right)-\\ & 2\left(h_{it}-o_{it}\right)\left(l_{it}-o_{it}\right)]-0.383{\left(c_{it}-o_{it}\right)}^{2}, \end{array}$$ (9)

where *o*_*it*_, *h*_*it*_, *l*_*it*_, *c*_*it*_ respectively stand for logarithm value of the opening, highest, lowest and closing price of the listed financial institution *i* at time *t*.

### Descriptive statistics of variables

**Fig 1:**
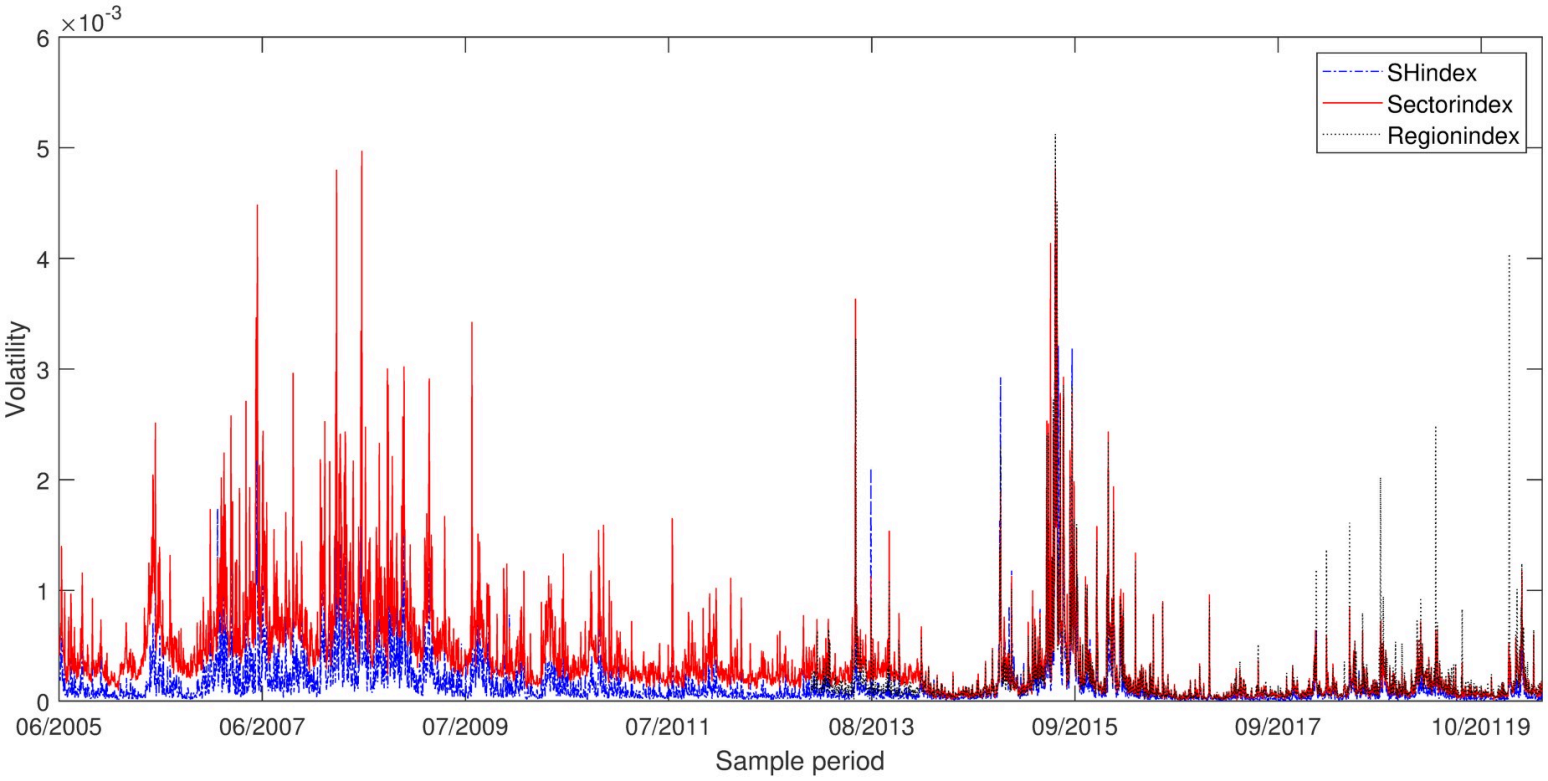
Comparison of volatility. Daily return volatility for Shanghai composite index(red), industry index(blue) and region index(dark).

We can respectively get daily return volatility for Shanghai composite index, industry index and region index based on Eq (9), and they are demonstrated in Fig 1. The volatility of industry index and region index have similar trend with the volatility of Shanghai composite index. The detailed description of volatility of indexes are presented in Table 1. However, the distributions of volatility have significant differences of certain degree of skewness and serious kurtosis. According to the combination of the information in Fig 1 and Table 1, it is not difficult to find that the volatility of the stock market has the characteristics of high peak and heavy tail, and the volatility is agglomerative. By explanation, the less larger volatility follows the large volatility, and smaller volatility follows small volatility.

**Table 1:** Description of indexes’ volatility.

Index	Average Volatility	Standard Error	Skewness	Kurtosis	Maximum Value	Minimum Value
Shanghai Composite Index	0.000168	0.000303	6.0575	56.2694	0.0041	0.00000257
Industry Index	0.000381	0.000447	3.7111	24.6196	0.005	0.00000886
Region Index	0.000197	0.000364	6.5308	60.4881	0.0051	0.0000113

Since 2005, China’s stock market has experienced two rounds of notable bullish and bearish. On June 1, 2005, the stock market closed at 1039.1870 points. Since then, the stock index gradually went up, and rose to 6124.044 points on October 17, 2007, reaching the peak of the bull market, and the index rose six times. However, the index then began to jump, and the US subprime mortgage crisis in 2008 further accelerate the downward trend of China’s stock market. Since then, China’s stock market entered the long-term downturn. In September 2014, the stock index rose gradually again and reached 5146.9490 points on June 8, 2015. But the China Securities Regulatory Commission prohibited securities companies from providing convenience for over-the-counter capital allocation, the stock index plummeted. After another round of bull market to bear market, the market entered a long recovery period. To compare the difference between risng market and falling market, we construct stock networks over different periods according to the stock market index fluctuation. The description above illustrates that the research period includes two rising periods and two falling periods. From June 2005 to May 2020, there are totally 3649 consecutive trading daily price. We divide the whole sampling period into four stages, including bullish trend(06/2005–10/2007), downturn(10/2007–03/2014), rapid increase(03/2014–06/2015) and falling(06/2015–05/2020) over the sample period.

### Parameter setting

In the process of the implementation of PMIME, several free parameters need to be identified, namely, the maximum time lags for each variable(*L*_*X*_), the threshold in the termination criterion, *T* representing the time ahead of time horizon, and the choice of these parameters depends on the specificity of dynamics. Since our observations are discontinuous time series, choosing a small lag in the process of implementation is much efficient but a larger lag is at the cost of unnecessary computations. According to [42, 43], *L*_*max*_ = 5 is set for all variables in view of the case. Besides, the threshold, an inherent parameter of PMIME, should work well for true direct couplings. According to a simulation study [46], the significance level for the termination is set as *α* = 0.05. The number of nearest neighbors for the estimation is *k* = 5, which is considerably stable. In addition, the choice of future time *T* is also dependent on dynamics, *T* = 1 is widely applied in linear and nonlinear causality measures in case of discontinuously sampled time series.

## Results and discussions

### Topology of industry index networks

**Fig 2:**
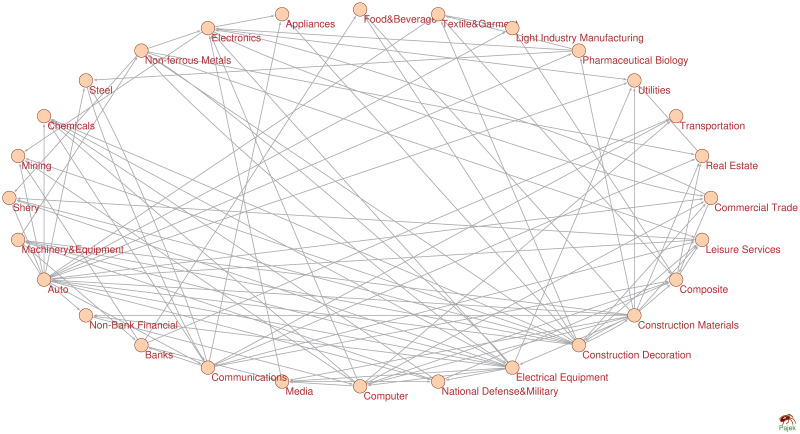
Industry index network. The industry index network is constructed by using PMIME over segment of June 2005–October 2007.

**Fig 3:**
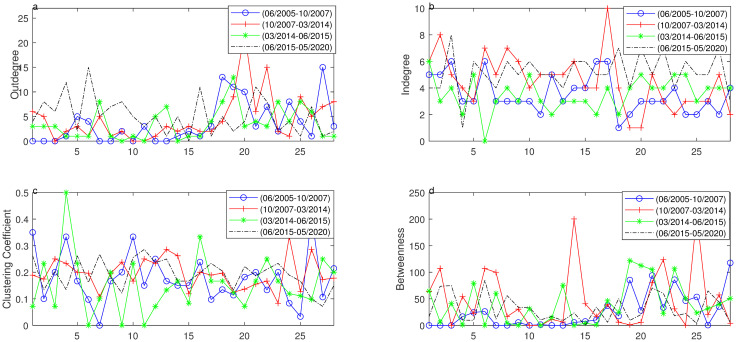
The topology of networks based on 28 industry sectors over four segments. The curve with circle represents the first segment, the curve with cross represents the second segment, the curve with asterisk represents the third segment and dotted curve represents fourth segment. **a**–**d** are respective distributions of outdegree, indegree, clustering coefficient and betweenness over four segments.

In view of network topology, an adjacency matrix *A*_*m*×*m*_ is calculated for industry index network by PMIME, in which *a*_*i*,*j*_ means the interaction from node *i* to *j* and *m* is the number of nodes in the network. We can find that the adjacency matrix is unsymmetric as the connections in the network represent directed effects. Therefore, the topology of directed network is different from undirected network. We construct industry index networks over four segments using PMIME (Fig 2, S1–S3 Figs). Fig 3 and Table 2 demonstrates networks topologies over four segments.

**Table 2:** Results of industry index over four segments.

Segment	Outdegree	Std(Out)^a^	Std(In)^a^	Clustering Coefficient	Distance	Betweenness
06/2005–10/2007	3.5357	4.2468	1.4006	0.1774	2.7897	26.1429
10/2007–03/2014	4.5000	4.8496	2.0458	0.1945	3.0044	48.3214
03/2014–06/2015	3.5357	3.2601	1.2615	0.1543	2.7875	39.9643
06/2015–05/2020	5.1786	3.7914	1.4415	0.2011	2.1975	37.1786

^*a*^std(Out) and std(In) are respective standard deviation of outdegree distribution and indegree distribution.

Degree, a common statistics of network, is classified into outdegree and indegree in directed graph. Outdegree means risk diffusion while indegree means absorption. The outdegree distribution differs widely over four segments, especially during the falling segments. Therefore, most sector nodes absorb the risk while only a few nodes play a role to transit risk. In another words, only a few nodes are driving nodes and have strong influences during falling segments. Compared with the first falling segment, the second falling segment witnesses a moderate increase in mutual interaction. As is shown in Fig 3, different driving nodes in varied segments, such as Construction Materials, Construction Decoration Materials, Electrical Equipment are strong over all segments while the extent is less strong in sectors like Computer, Non-Bank Financial, Auto, National Defense & Military. With respect to indegree, the changes can be seen over four segments with wide range of fluctuations in falling segments. Most nodes are prone to be affected, among which Composite, Chemicals and Shery sectors are more easily affected compared with other nodes.

The clustering coefficient has varied from 0 to 0.5 over four segments, during which the falling segments have a bit higher extent of clustering and slight changes among all sectors. By contrary, the clustering coefficient has large fluctuation over the first and third segment, and even some sectors have no clustering effect. Especially, the sectors of Shery, Steel, Non-ferrous Metals, Transportation, Leisure Services, Construction Materials, National Defense & Military, Computer and Auto have higer level of clustering effect.

In the distance of four segments, the average distances (except unreachable pairs), ranging from 2.1975 to 3.0044, have slight differences. In these four segments, there are similar distances in two rising periods but an increase is detected during the first downturn period and the distance in the fourth segment is smallest. With the development of stock market, the more interactions are found, so the information transmission is faster in the fourth segment compared with the second segment.

Based on the distance, we can get the betweenness of each sector over four segments. The significant changes of betweenness can be seen over the second and fourth segment, especially during the second segment. Since more interactions have been found in the network, each segment has similar betweenness over the fourth segment while there is no betweenness in some sectors over the first three segments. The sectors of Construction Decoration Materials, Electrical Equipment, Communication, Auto have larger betweenness over rising segments while sectors of Mining, Electronics, Real Estate and Machinery & Equipment have larger betweenness over falling segments. And National Defense & Military sector has the largest betweenness over all segments.

In the varied segments, the number of edges in the network are respectively 99, 126, 99 and 145, about 3*m* − 5*m*(*m* is the amount of nodes in the network). In the first period, there are 7 4–*clique* and 18 3–*clique*. In the second period, there are only 5–*clique*, 11 4–*clique* and 19 3–*clique*. In the third period, the number of 4–*clique* is 4 and 3–*clique* is 16. In the fourth period, more cliques are formed in terms of 1 6–*clique*, 3 5–*clique*, 8 4–*clique* and 15 3–*clique*. Compared with PMFG, it can only supply cliques constructed with 3 nodes and 4 nodes by connecting 3(*m*–2) edges. Using PMIME, as the number of cliques varies with time, fewer cliques are formed during rising time while more and larger cliques are constructed during the falling periods because mutual interactions get more.

**Fig 4:**
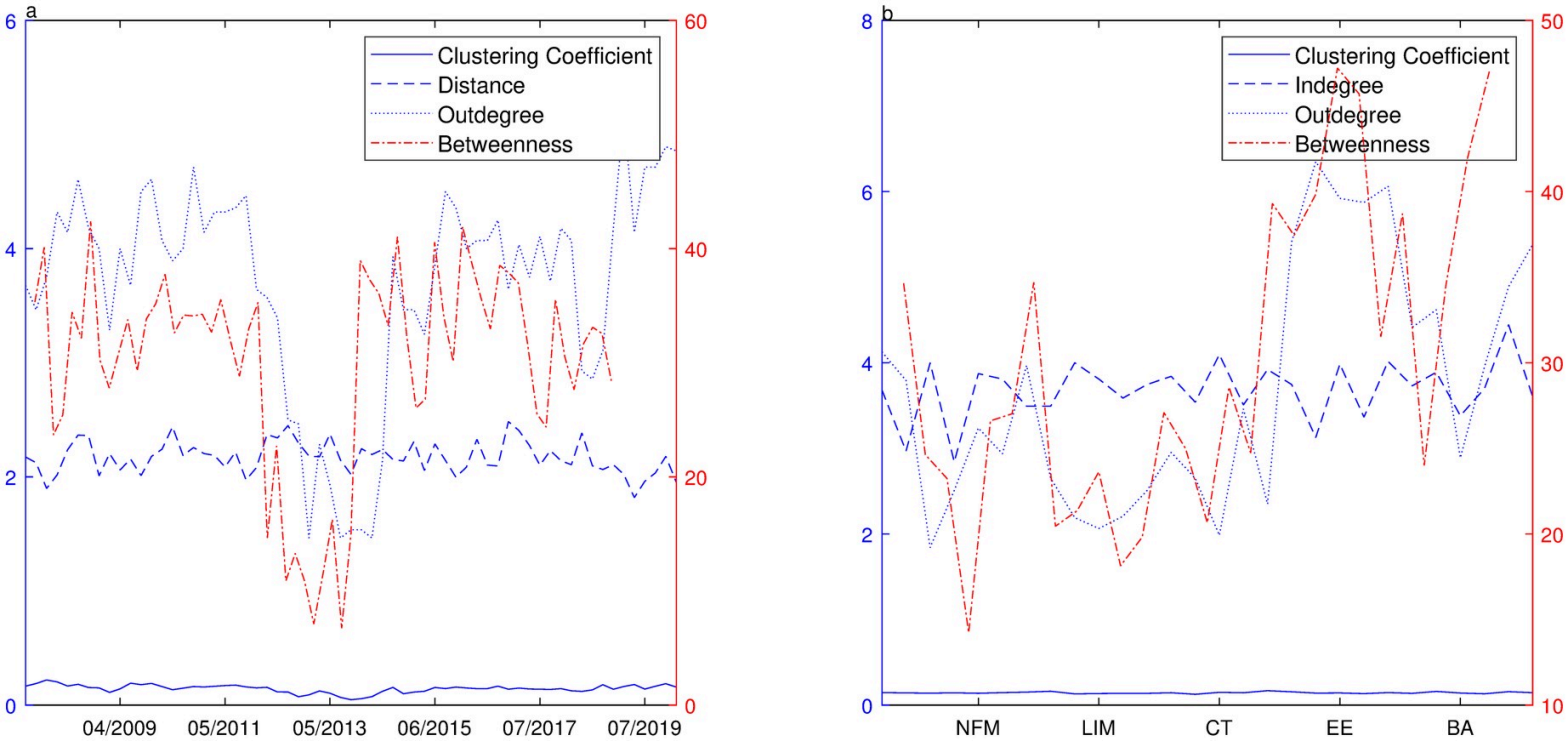
Network topology of industry index over rolling windows. **a** Shows distributions of the clustering coefficient(solid curve), the distance(dashed curve), the outdegree(dotted curve) and the betweenness(dash-dot curve) over different periods where the clustering coefficient, distance and outdegree are referred to the left Y-axis and average betweenness is referred to the right Y-axis. **b** Shows distributions of clustering coefficient(solid curve), indegree(dashed curve), outdegree(dotted curve) and betweenness(dash-dot curve) for each industry sector during the whole period where the clustering coefficient, the indegree and the outdegree are referred to the left Y-axis and the betweenness is referred to the right Y-axis.

In order to trace the relationship between risk spillover and topological changes of the security market, we will take more segments into consideration. The appropriate time series length should not be less than 250 since the setting *n* = 250 is about trading days in a year. Considering the embedding vector and nonlinear relationship, we think that *n* = 500 (about two years) may be the proper length for estimation. We select a rolling window of 50 days(almost two months) and make the detailed comparison from the perspective of time and varied sectors. From the perspective of time, average indegree is equal to average outdegree, so we do not report average indegree in Fig 4a. In Fig 4b, average indegree distribution is different in each sector and is correlated to risk input, so we report it. And the distance for some sectors is unreachable, then this statistics is omitted in Fig 4b.

The outdegree distribution fluctuates obviously both over the varied periods or on identical sectors, ranging from 1.2500 to 5.6071 over the varied periods, and an increase of the outdegree is observed during the rapid rising and slumping periods. The mean outdegree for each sector shows that outstanding driving sectors are Construction Materials, Construction Decoration Materials, Electrical Equipment, Computer, National Defense & Military, Communications, Machinery & Equipment and Auto. These industries can be regarded as risk output since their fluctuations can affect other industries significantly. With respect to indegree, sectors of Chemicals, Textile and Garment, Light Industry Manufacturing, Commercial Trade and Machinery & Equipment are more likely to be affected, so they are regarded as risk absorption sectors. It is noteworthy that some industries are the type of both risk output and input, such as Machinery & Equipment, Communications. According to the calculation, the distribution of average distance is relative stable over the whole period, about 2–2.5 steps among reachable pairs. The distribution of betweenness is quite consistent with the distribution of outdegree. It gets larger during the rapid rising and slumping periods. Averagely, the sectors of Construction Materials, Electrical Equipment, National Defense & Military, Computer, Machinery & Equipment, Auto have larger betweenness. To investigate the risk property of varied sectors, we classify nodes into four types, risk-outgoing, risk-incoming, risk-bridging and risk-bordering, considering each sector has different role in the process of risk propagation. According to Eqs (7) and (8), the average outdegree centrality falls in [0.1104, 0.5105] and only 21% of sectors are over 0.4. The average indegree centrality falls in [0.2461, 0.5480] and 21% are over 0.5. It is observed that the outdegree and indegree centrality are not equal in each sector. Then these sectors are divided into different types and Table 3 shows the results of classification. Among all these sectors, only a few nodes are risk-output type and most nodes are risk-input type. Still some nodes have function of both risk-output and risk-input, called bridging nodes while only one node has weak connections with other nodes, called bordering node.

**Table 3:** Classification of nodes.

Type	Sectors
Risk Outgoing(only *OCI* ≥ 0.4)	CM, CDM, EE, NDM, CPT, MED, AU
Risk Incoming(only *ICI* ≥ 0.5)	MI, CHE, NFM, FB, ELE, HA, CT, LIM, TG, PB, UT, CP, STE, RE, TS, LS
Risk Bridging(*OCI* ≥ 0.3&*ICI* ≥ 0.4)	SY, ME, CMM, NBF
Risk Bordering(*OCI* < 0.3&*ICI* < 0.3)	BA

**Fig 5:**
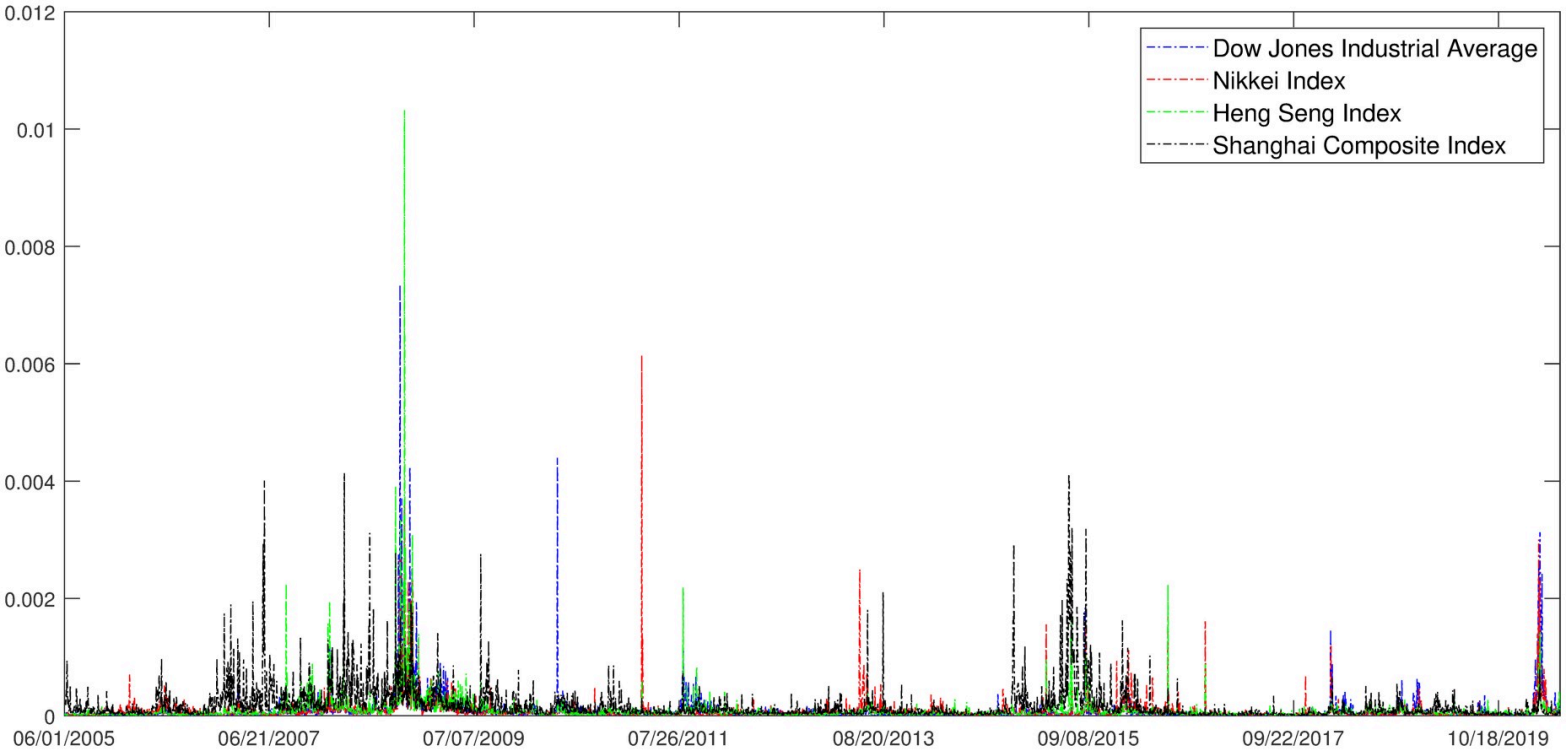
Comparison of volatilities in four stock markets. Daily return volatility for Dow Jones Industrial Average Index(blue), Nikkei Index(red), Heng Seng Index(green) and Shanghai Composite Index(dark).

Due to the development of economic globalization, the stock markets over the world have stronger interactions than before. In order to trace the mutual influence among other security markets and China’s security market. We have selected three indexes including Dow Jones Industrial Average Index, Nikkei Index, Heng Seng Index, which are strongly correlated to the China’s stock market. As shown in Fig 5, four distinct market indexes have the common characteristic that the fluctuations are clustered over the same segments and are relatively intensive.

**Fig 6:**
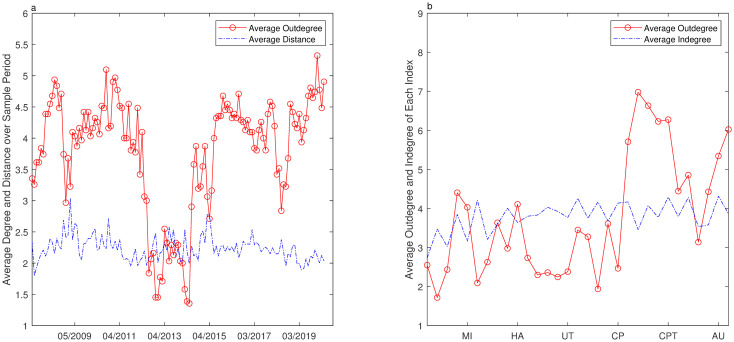
Network topology of industry index over rolling windows. **a** Shows distributions of outdegree and indegree over different periods. **b** Shows outdegree and indegree for different indexes during the whole period.

A mixed network has been constructed by the 3 international indexes and 28 industrial indexes. By selecting a rolling window of 24 days(almost trading days in a month) over the whole sample period, we get topologies of network from the perspective of time and varied indexes (Fig 6). According to the topology of the mixed network, the indexes could be distinctly divided into two groups which are market indexes and industrial indexes. Averagely, these three market indexes have more directed links with each other and less with the 28 industry indexes. Among the three market indexes, Dow Jones Industrial Average has the biggest outdegree and Nikkei Index has the second biggest outdegree. Therefore, the impact mechanism of overseas stock markets on China’s stock market is first transmitted to some sectors and then to the whole market. Over the whole sample period, the volatility of Dow Jones Industrial Average has mainly affected industrial sectors such as Electronics, National Defense & Military, Computer and Machinery & Equipment. The volatility of Nikkei Index can cause the significant fluctuations of industrial sectors such as Household & Appliance and Light Industry Manufacturing. The Heng Seng Index has more direct influence on industrial sectors, especially on Steel, Food & Beverage, Computer, Communications and Machinery & Equipment. But these 28 industry indexes do not have much directed influence on other market indexes. The degree distributions among 28 industrial indexes are similar to the results above. The distribution of distance is similar to that of outdegree, but the volatility is small.

### Topology of region index networks

We collect region indexes of 1800 consecutive trading days from December 2012 to May 2020. Similarly, there have been three subsamples in the sample of region indexes whose period are divided as downturn period(12/2012–03/2014), rapid increase period(03/2014–06/2015) and downturn period(06/2015–05/2020). Then we study networks (S4–S7 Figs) of the whole period and three divided periods by using PMIME.

Based on PMIME, we get results of three segments and over the whole period. Because the first period(12/2012- 03/2014) is the beginning of region index compilation, less information is revealed. Therefore, we report the results for the whole period, the second and the third period (Table 4). The outdegree of the third period is largest, ranging from 0 to 19, but only 4 region nodes have a degree of more than 10. In the long run, the average outdegree is 5.2222, smaller than that of falling period. The indegree and distance distributions have slight changes over three periods. In the third period, clustering coefficient is the largest but betweenness is the smallest. Region nodes have more connections in the falling period but only a few nodes act as a way to convey information. The falling period has the smallest betweenness but the largest connections in the network, where most nodes are risk absorbent. Therefore, the topology of region index network is similar to the industry index network. The information transmission is effective in the rising period while more connections are found in the downturn period.

**Table 4:** Results of region index over three periods.

Segment	Outdegree	Std(out)	Std(in)	Distance	Clustering Coefficient	Betweenness
12/2012–05/2020	5.2222	3.5141	1.3961	2.2524	0.1467	44.8056
03/2014–06/2015	4.4722	3.2992	1.1829	2.3746	0.1396	50.0556
06/2015–05/2020	5.5000	4.4175	1.3202	2.2524	0.1797	39.4722

The networks are respective constructed by 161 and 198 edges over the rising and downturn periods. During the rising period, there are 7 4-clique and 21 3-clique while 2 5-clique, 12 4-clique and 21 3-clique are formed during the downturn period. We found that there are fewer cliques during rising time but more and larger cliques during down turn period. This result is similar to the situation of industry index cliques, which display Shenzhen Enterprises Composite, Bohai, Qinghai, Chongqing and Shaanxi have more cliques during the rising period while Shenzhen Inno, Yangtze, Guizhou, Inner Mongo, Ningxia have more cliques.

**Fig 7:**
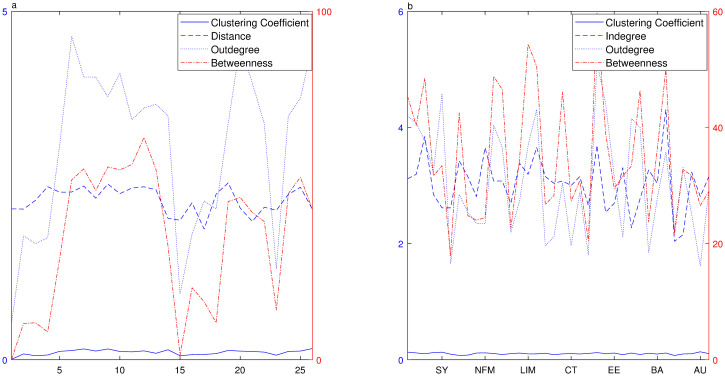
Network topology of region index over rolling windows. **a** Shows distributions of the clustering coefficient(solid curve), the distance(dashed curve), the outdegree(dotted curve) and the betweenness(dash-dot curve) over different periods where the clustering coefficient, distance and outdegree are referred to the left Y-axis and average betweenness is referred to the right Y-axis. **b** Shows distributions of clustering coefficient(solid curve), indegree(dashed curve), outdegree(dotted curve) and betweenness(dash-dot curve) for each industry sector during the whole period where the clustering coefficient, the indegree and the outdegree are referred to the left Y-axis and the betweenness is referred to the right Y-axis.

To study the dynamic relationship among region sectors, we take *n* = 300 as a segment length and 60 days as a sliding window(Fig 7). In terms of time, the mean outdegree varies violently over different segments, ranging from 0.5278 to 4.6389, and it is higher during the period when the market index fluctuates intensely. The change of betweenness is quite consistent with the distribution of outdegree, and it gets larger during the rapid rising and slump periods. The averaged distance among reachable pairs and clustering coefficient distribution are quite stable. From the region perspective, the distributions of outdegree, indegree and betweenness have large fluctuations, showing that each region plays a different role in the network. According to Eqs (7) and (8), the average outdegree centrality takes value in [0.1200, 0.4147] while the average indegree centrality takes value in [0.2524, 0.6468]. It is a bit different from industry sector network, the region sectors are only classified into types of risk incoming and bridging. The regions sectors of Shenzhen Innovation, Shenzhen Enterprises Composite, Yangtze, Bohai, Beijing, Shanxi, Inner Mongo and Shanghai are the type of risk bridging while the other region sectors are the type of risk incoming. From the perspective of region sectors, only a few region sectors have strong influence on other sectors, meanwhile they are influenced by other sectors as well. Most sectors are likely to be influenced, consistent with China’s regional economic development.

### Robustness test

Since the rising period is longer than the downturn period in China’s security market, the result based on the measure PMIME might be affected by the length of time series. In order to test the robustness of measure PMIME, we further divide the decrease segment into slumping and downturn segments according to the fluctuation of market index. Then the whole period is divided into 6 segments, namely, June 2005-October 2007(rising), November 2007- March 2010(slumping), April 2010–August 2012(downturn), February 2013–June 2015(skyrocket), July 2015–November 2017(slumping) and December 2017–April 2020(downturn). As is illustrated in Table 5, the slumping segments are discovered to have more interactions than rising and downturn segments and the latest slumping segment has the most interactions. For the segments with the same trend, the latest one has more interactions. During the segment of April 2010–August 2012, the difference of indegree is the largest of all. These results are consistent with the results above, so the measure PMIME can better explore the interactions among sectors over varied segments and is robust in the respect of the length of time series.

**Table 5:** Results of industry index over six segments.

Segment	PMIME	Outdegree	Std(Out)	Std(In)	Distance	Edges
06/2005–10/2007	0.1352	3.5357	4.3600	1.4006	2.7897	99
11/2007–03/2010	0.1248	4.2800	4.1789	1.3012	2.3597	120
04/2010–08/2012	0.2041	3.9643	3.5011	2.2523	2.0528	111
03/2013–06/2015	0.1431	4.0714	3.1848	1.3313	2.2825	114
07/2015–11/2017	0.1025	4.8929	2.9230	2.0965	2.1429	137
12/2017–04/2020	0.1540	4.2500	3.6780	1.2360	2.2531	119

### Discussion

The findings of empirical analysis from above provide evidence that the networks generated by PMIME reveal dissimilar topologies over different segments, which can indicate the financial risk spillover through the interaction of fluctuations. The identical levels of the risk spillover effect over different segments, showing that the structure during the bear market is very different from that during the bull market. During the rising stage, the response of the market is relatively effective and the interaction of fluctuations between varied sectors are very active and rapid. But during the decline stage, the interaction of fluctuations seems to be more complicated, there are more connections and the great difference in degree distributions of nodes. The strengthening of risk spillover among sectors will lead to the increase of network density in the process of market collapse, which is consistent with research [51]. China’s stock market has been driven by capital in the process of rising, with all sectors rising sooner or later. In the process of falling, the withdrawal of funds from the market, coupled with the impact of market sentiment. Since panic sentiments are not easy to be digested by the financial market, the ineraction of fluctuations would last for a long time and all stocks fall in response. During the recovery period following the rapid decline, market performance is most correlated with economic fundamentals.

In each network, only a few nodes have higher degree while most nodes have lower degree. Therefore, we can find that both industry indexes and region indexes networks have similarities that all sectors perform significant price fluctuations over varied periods but only a few sectors dominate the market and only a few sectors have essential roles in the network while most sectors are influenced. This demonstrates that the network becomes local centralized, which is mainly reflected in the risk diffusion effect of some crucial nodes and the importance of their neighbors. The centralization of network makes the systemic risk greatly depend on the risk status of individual nodes. The nodes in the connected position are forced to fluctuate with the fluctuation of the central nodes. In the long term, the connection between sector nodes increase and the characteristics of risk spillover become more prominent. For industry index network, only a few sectors are risk outgoing while most sectors are risk incoming. But for region index network, most region nodes are both of risk outgoing and incoming. From the perspective of time, more connections are significantly presented during the latest period and fewer connections at the beginning since we get samples over the comparable long time, which means the complexity of financial system has been considerably increasing. When the risk spreads through the nonlinear correlation of stocks in the network, it causes a chain reaction and finally infects the whole network.

Considering the risk spillover from international markets, China’s security market has been closely linked to the markets from US, Japan and Hongkong. Among these three security markets, Dow Jones Stock Index has the strongest power, which has been considered to be a central place in the world compared to the other two indexes. Therefore, Dow Jone Stock Index has played a predictive role in the performance of global security markets. And Nikkei Index has less power while Heng Seng Index has least influence to China’s security market. However, the risk spillover from Dow Jone Stock Index has transfered through only a few industry indexes of China’s security. By comparison, the volatility of Heng Seng Index can cause more response of industry indexes.

We make comparison of PTE and PMIME by using sample of industry index. Several free parameters should identified, embedding dimension *m* = 5, time step ahead *T* = 1, the number of nearest neighbors for the estimation *k* = 5. We study the nonlinear correlation among industry sectors over four segments of bullish trend(06/2005–10/2007), downturn(10/ 2007–03/2014), rapid increase(03/2014–06/2015) and falling(06/2015–05/2020). As is illustrated in Table 6, the results are similar to those of the PMIME. The distribution of outdegree is not uneven while the distribution of indegree has fewer differences. The falling segments have more connections compared with rising segments. The networks constructed by PTE and PMIME have a few differences on some connections, since the PMIME takes the lagged variables into considerations. Concerning the measures, the PMIME outperforms the PTE. The PTE fails to detect the relationship with lagged variables. And the PMIME suits to the large data set and high dimensional case, although there might be slightly different number of connections when applying different length of rolling window. In real applications, especially in financial time series, stock price fluctuations have characteristics of the time lag.

**Table 6:** Results of industry index over four segments based on PTE.

Segment	Outdegree	Std(Out)	Std(In)	Clustering Coefficient	Distance	Betweenness
06/2005–10/2007	3.7143	4.3449	1.4620	0.1930	2.2531	25.1786
10/2007–03/2014	4.5357	4.9701	1.5512	0.1941	2.2156	36.6786
03/2014–06/2015	3.5000	3.1091	1.1386	0.1457	2.3095	41.2143
06/2015–05/2020	5.1786	3.7024	1.0905	0.2003	2.0608	29.6071

## Conclusion

The measure of PMIME has been utilized effectively in analysis of risk spillover in the financial market since it can better explain the nonlinear correlation. So far we have constructed industry index and region index networks based on this nonlinear measure and have made the comparison with the PTE. Through this explored measure, the interrelationships have been investigated between any two distinct vectors under the condition of embedding vectors and the direction of interrelationships has been specified. We can capture the risk source and make a better understanding of risk transmission in the stock market and the mechanism of risk spillover among diverse markets. Through the analysis, we found it is a fairly new area of research to develop the financial network. The financial system is fast evolved with the time, as our current work describes dynamic in the way of rolling windows and real-time evolution should be considered in the network in future. Furthermore, we should consider more market indicators instead of the only price index when we discuss the risk spillover effect in financial system, such as volume and volatility [52]. Therefore, our future work could focus on the construction of weighted directed network to precisely measure the risk transmission but under the condition that more indicators are involved.

## Supporting information

S1 Fig Industry index network. The industry index network is constructed by using PMIME over segment of October 2007–March 2014.
(TIF)

S2 Fig Industry index network. The industry index network is constructed by using PMIME over segment of March 2014–June 2015.
(TIF)

S3 Fig Industry index network. The industry index network is constructed by using PMIME over segment of June 2015–May 2020.
(TIF)

S4 Fig Region index network. The region index network is constructed by using PMIME over period of December 2012–May 2020.
(TIF)

S5 Fig Region index network. The region index network is constructed by using PMIME over period of December 2012–March 2014.
(TIF)

S6 Fig Region index network. The region index network is constructed by using PMIME over period of March 2014–June 2015.
(TIF)

S7 Fig Region index network. The region index network is constructed by using PMIME over period of June 2015–May 2020.
(TIF)

S1 Data (RAR)
