# Supplementary figures and images for: Risk spillover networks in financial system based on information theory
Source: PLoS One. 2021 Jun 18;16(6):e0252601. doi: 10.1371/journal.pone.0252601 (PMC8213145; doi:10.1371/journal.pone.0252601)

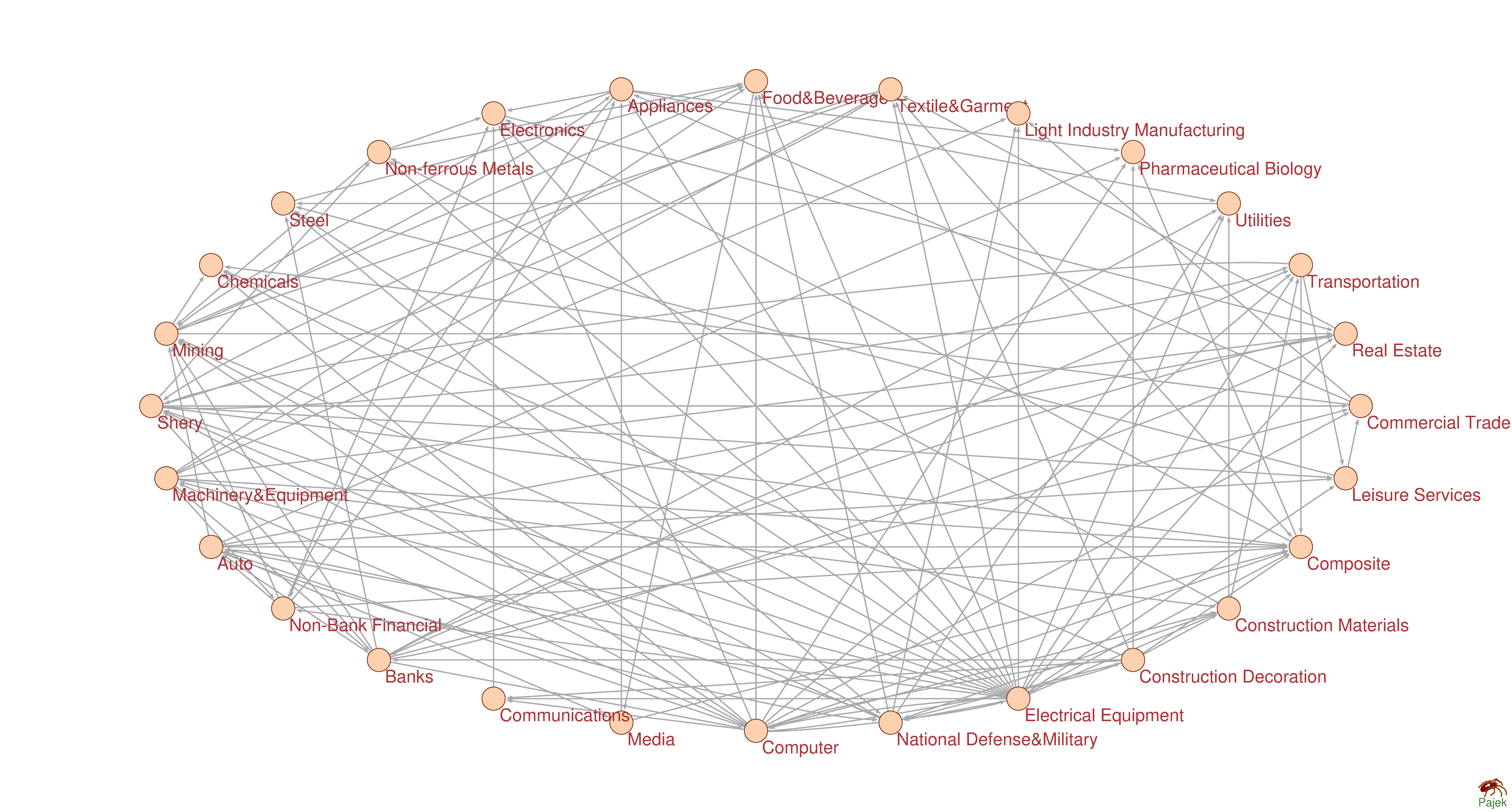

Supplement: S1 Fig — The industry index network is constructed by using PMIME over segment of October 2007–March 2014. (TIF) [file pone.0252601.s001.tif]

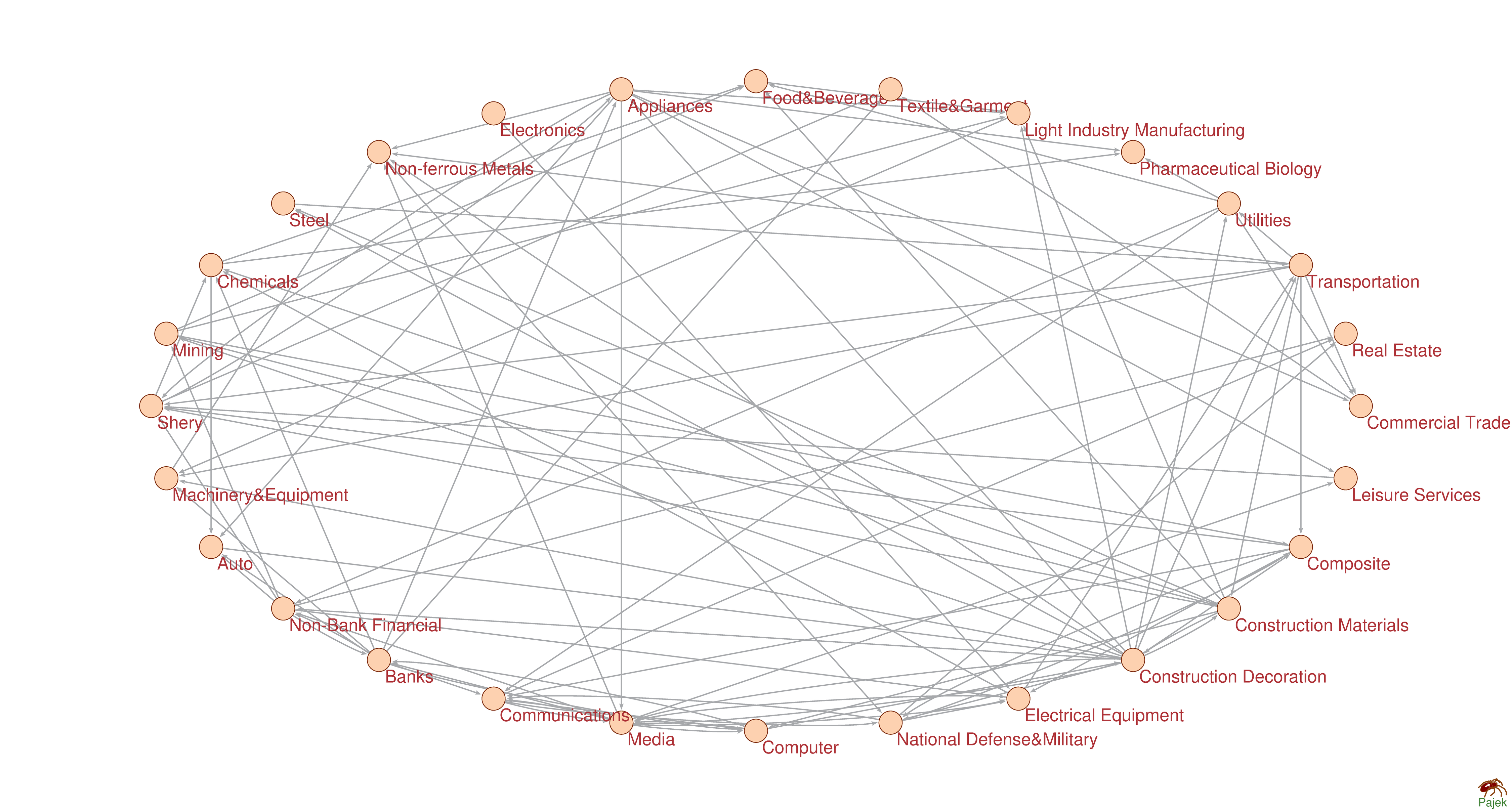

Supplement: S2 Fig — The industry index network is constructed by using PMIME over segment of March 2014–June 2015. (TIF) [file pone.0252601.s002.tif]

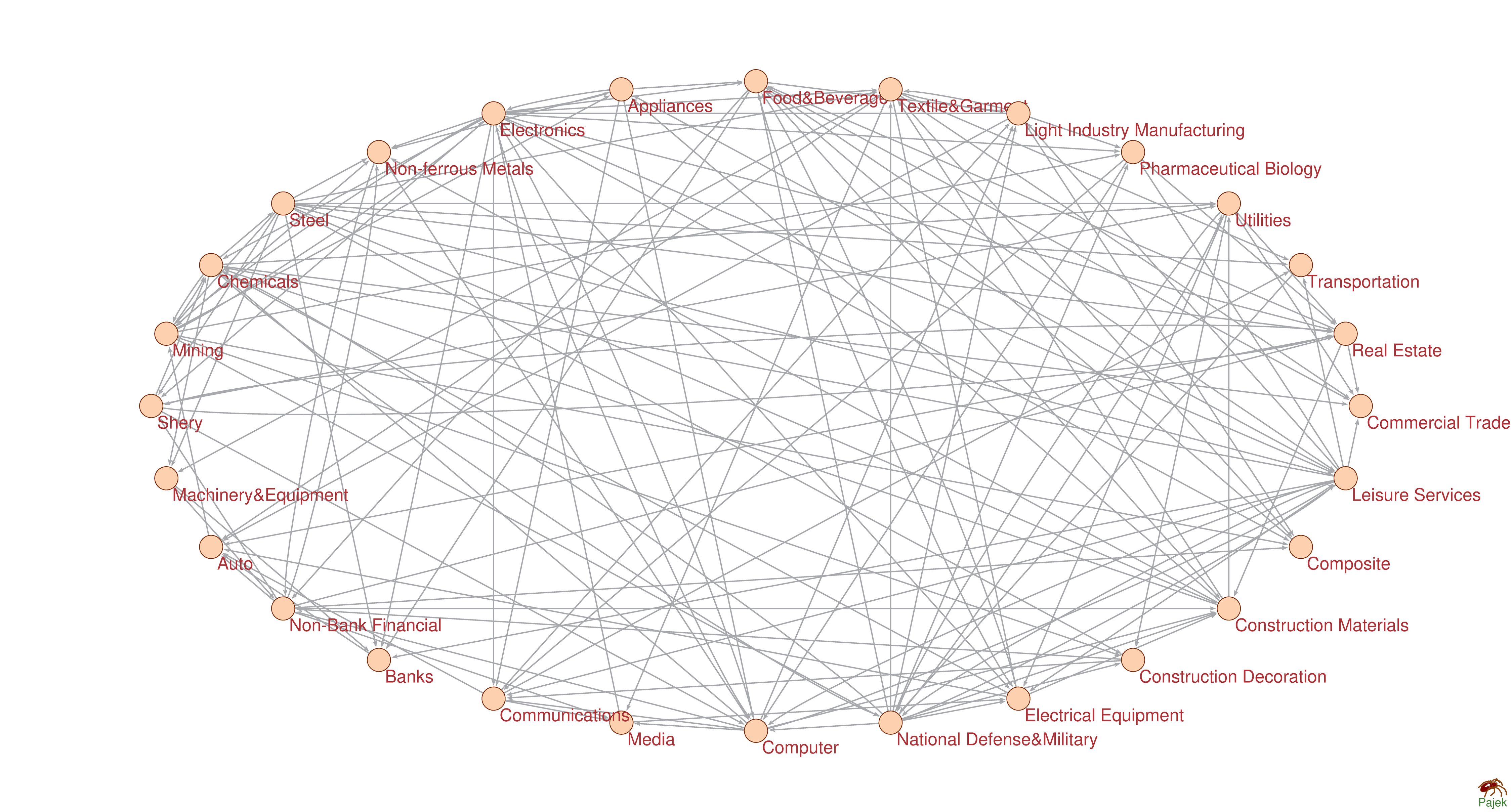

Supplement: S3 Fig — The industry index network is constructed by using PMIME over segment of June 2015–May 2020. (TIF) [file pone.0252601.s003.tif]

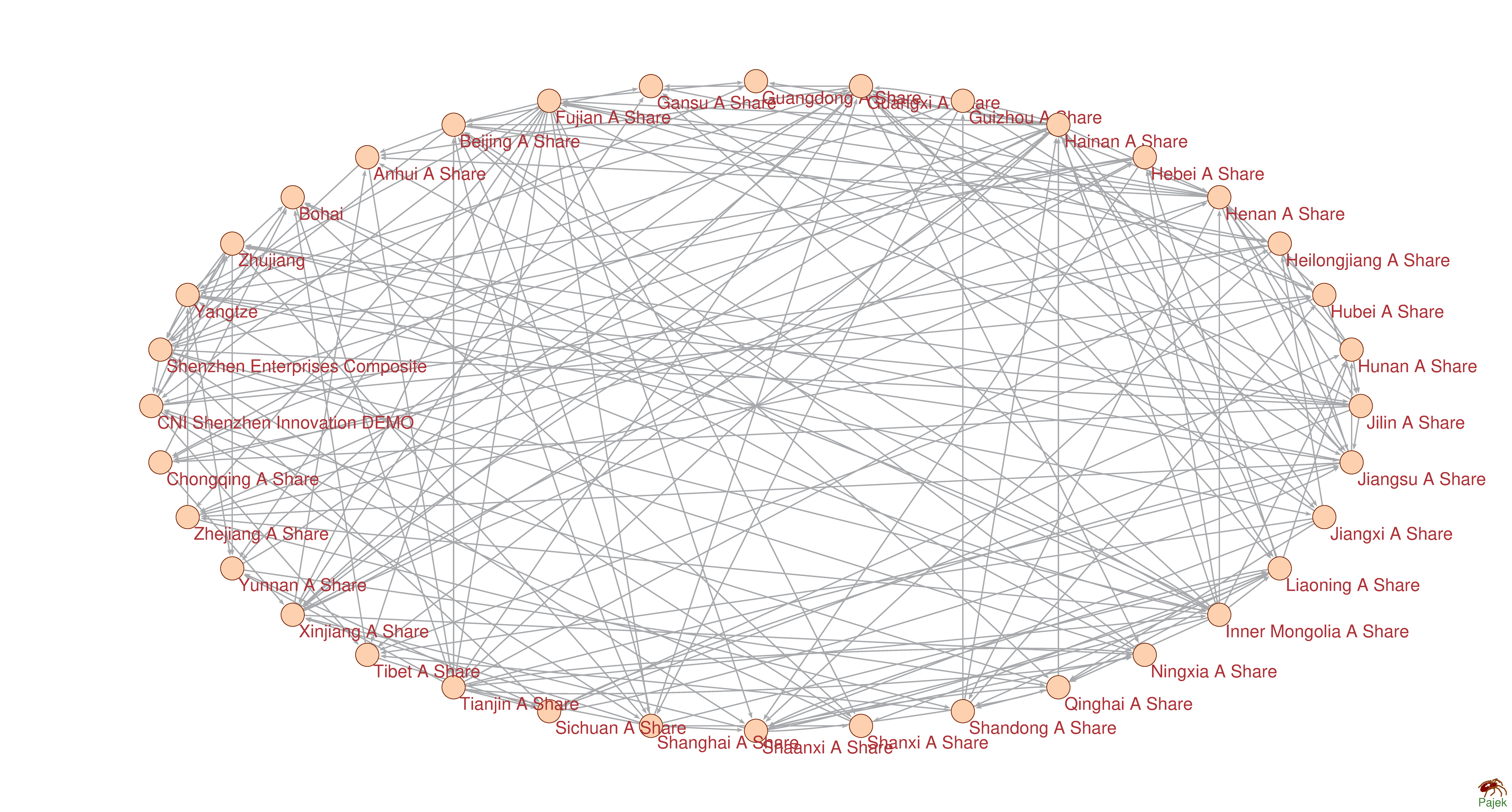

Supplement: S4 Fig — The region index network is constructed by using PMIME over period of December 2012–May 2020. (TIF) [file pone.0252601.s004.tif]

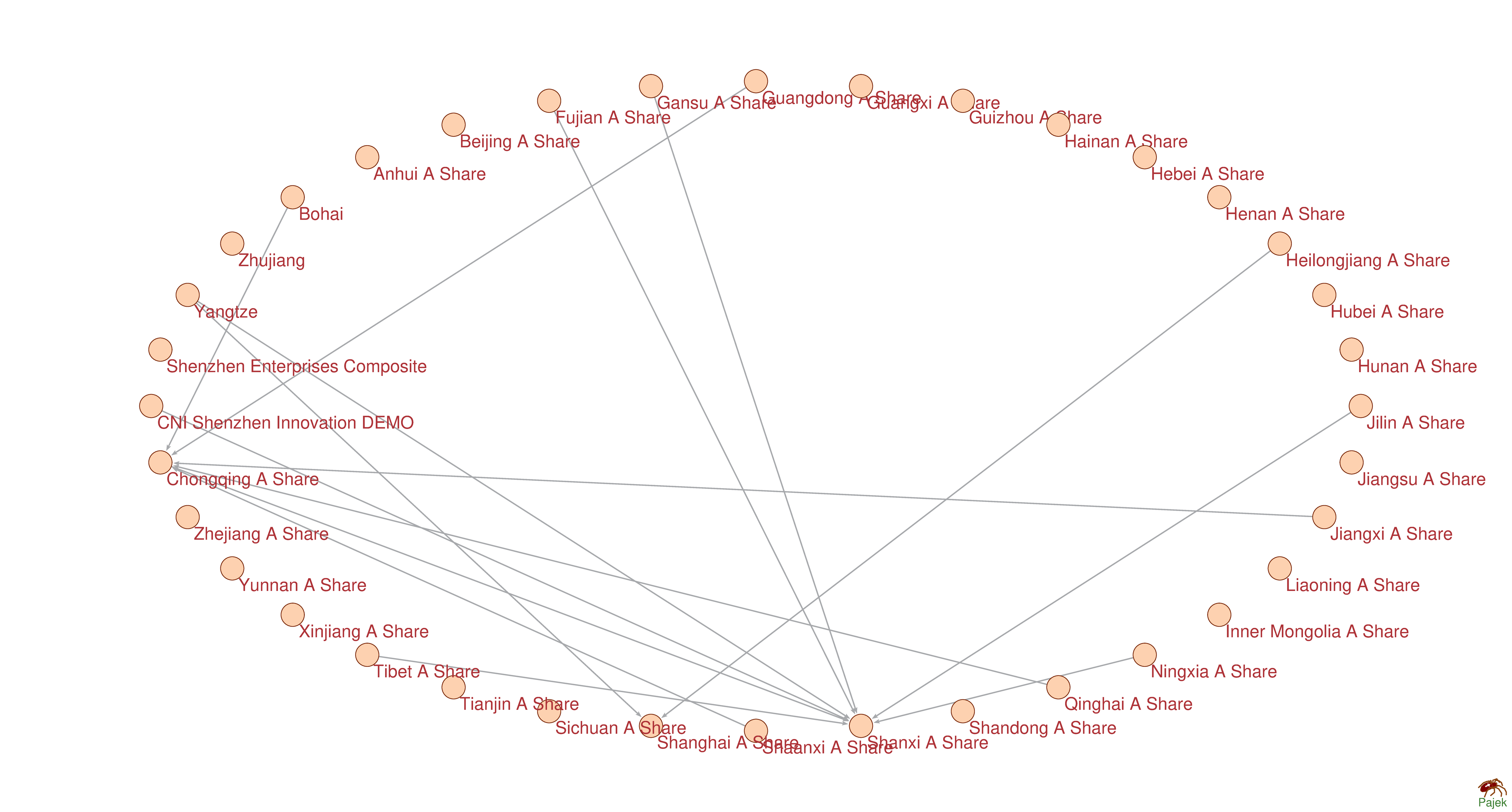

Supplement: S5 Fig — The region index network is constructed by using PMIME over period of December 2012–March 2014. (TIF) [file pone.0252601.s005.tif]

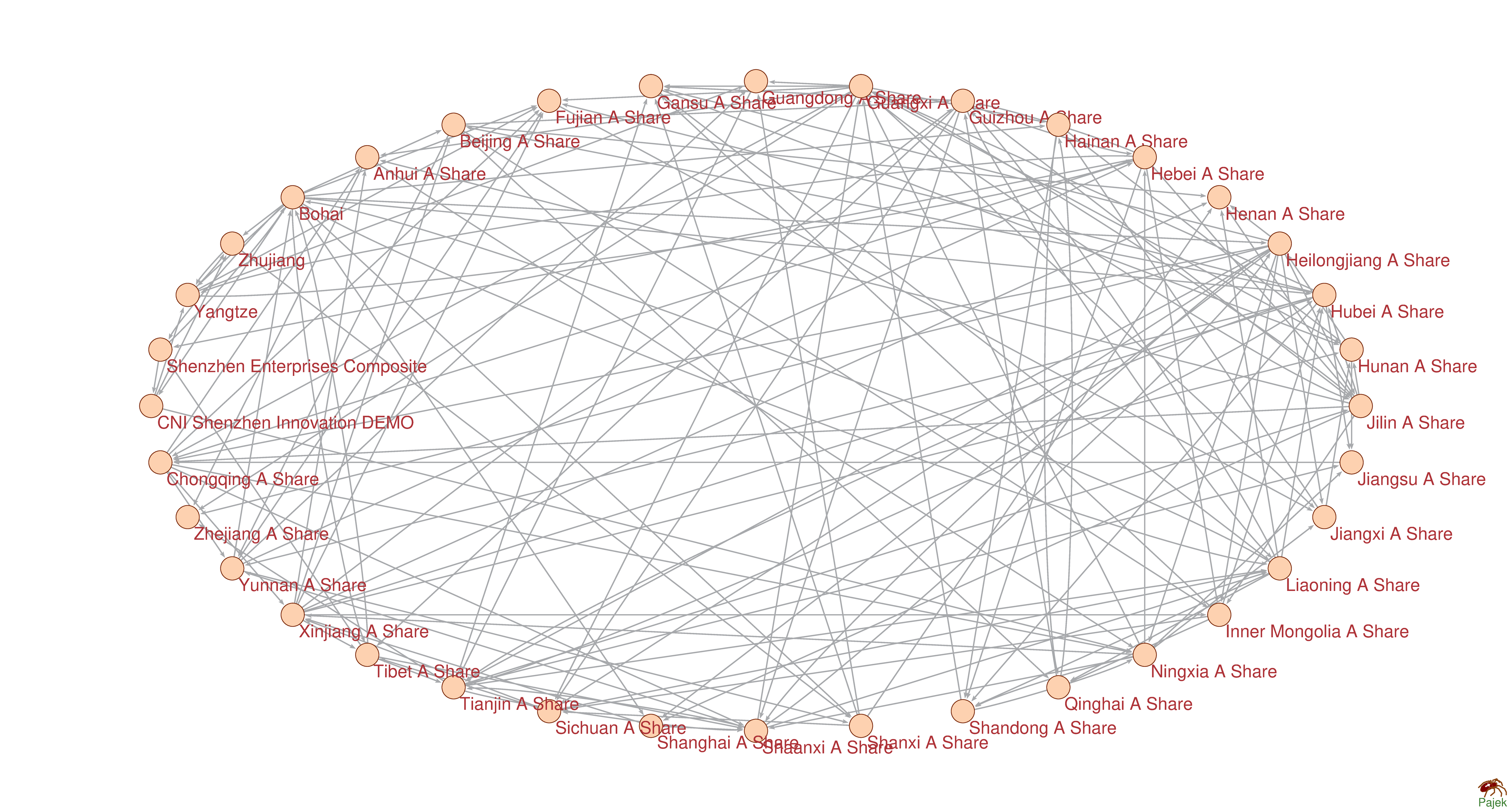

Supplement: S6 Fig — The region index network is constructed by using PMIME over period of March 2014–June 2015. (TIF) [file pone.0252601.s006.tif]

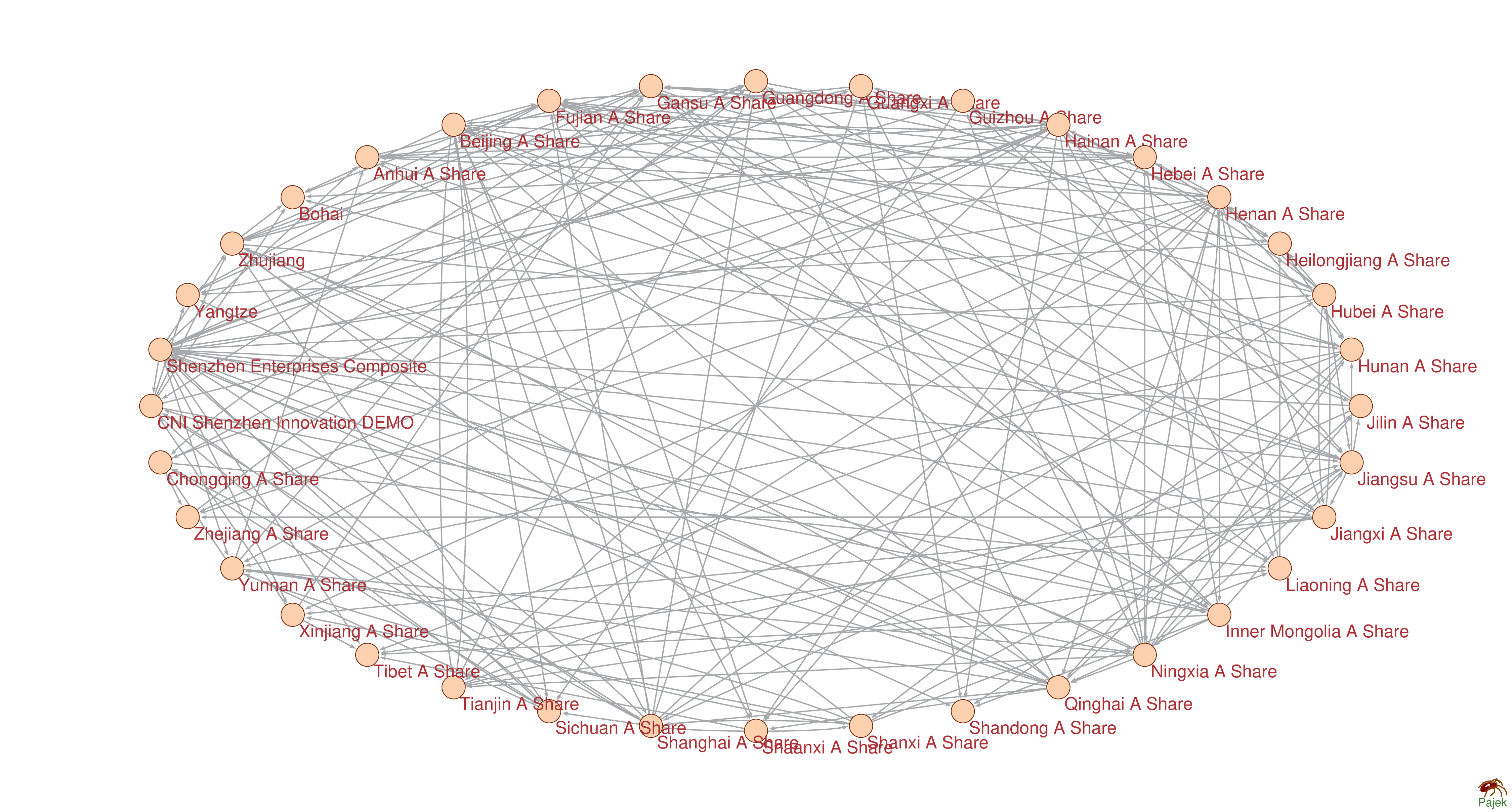

Supplement: S7 Fig — The region index network is constructed by using PMIME over period of June 2015–May 2020. (TIF) [file pone.0252601.s007.tif]
